# Parents’ Knowledge and Attitude towards HPV and HPV Vaccination in Poland

**DOI:** 10.3390/vaccines10020228

**Published:** 2022-02-02

**Authors:** Katarzyna Smolarczyk, Anna Duszewska, Slawomir Drozd, Slawomir Majewski

**Affiliations:** 1 Department of Dermatology Immunodermatology and Venereology, Medical University of Warsaw, 02-008 Warsaw, Poland; slawomir.majewski@wum.edu.pl; 2 Division of Histology and Embryology, Department of Morphological Sciences, Faculty of Veterinary Medicine, Warsaw University of Life Sciences, 02-776 Warsaw, Poland; duszewskaanna@hotmail.com; 3 Institute of Physical Culture Studies, College of Medical Sciences, University of Rzeszow, 35-959 Rzeszów, Poland; slawek.drozd@op.pl

**Keywords:** HPV, HPV vaccine, human papillomavirus

## Abstract

HPV is one of the diseases of civilization that causes cervical cancer, among other diseases. For this reason, a vaccination program has been introduced worldwide for preadolescent, sexually inactive seronegative girls. However, the decision to vaccinate young girls must be made by the parents. In Poland, vaccinations are recommended but not financed by the government, which affects their choices, and there is insufficient knowledge of the diseases caused by genital HPV types. In addition, there are cultural, social, and even religious factors to be considered. Therefore, the aim of the study was to analyze the state of knowledge about HPV and HPV vaccines among parents. Two hundred and eighty-eight parents participated in the study, but only 180 of them declared that they had ever heard of HPV (62.5%). Therefore, only these parents completed the entire questionnaire consisting of 34 questions. The parents’ answers were analyzed with the Fisher’s and chi-squared tests. The study showed that parents’ knowledge of HPV and HPV vaccination in Poland is low (49.4% of correct answers). Parents’ attitudes were only influenced by knowledge and education and not by other parameters such as age, gender, place of residence, and the number of children. This study indicates that parents need to be educated about the threats of HPV and the possibilities of prophylactic vaccination.

## 1. Introduction

Human papillomaviruses (HPVs) belong to the family *Papillomaviridae* and are non-enveloped icosahedral, circular, dsDNA viruses [1]. HPVs infect the cutaneous and mucosal epithelia, hence its wide spectrum of occurrence (skin, oral cavity, oropharynx, larynx, anogenital tract) [2]. There are more than 207 types of HPV [3], most of which do not cause any symptoms, lesions, or warts and are referred to as low-oncogenic (types). However, some types of HPVs are highly oncogenic and can induce intraepithelial neoplasia or cancers [2,4]. Among other diseases, cervical cancer is associated with types 16, 18, 31, 33, 35, 39, 45, 51, 52, 56, 58, 68, 73, 82, and especially 26, 53, and 66 [2]. In the European population, eight types are of particular importance (16, 18, 31, 33, 35, 45, 56, and 58), with 16 and 18 being responsible for 70 percent of all cases of cervical cancer [5].

In 2012, there were 530,000 cases of cervical cancer related to HPV, of which 370,000 were caused by HVP16/18 (71%) [6]. In addition, more than two-thirds of the cases are diagnosed in less developed countries [7,8]. Interestingly, the highest number of cases of HPV cervical cancer occurs in Asia (India 120,000) and Sub-Saharan Africa (93,000), and one of the lowest in Australia/New Zealand (940) [6]. The age-standardized mortality rate for cervical cancer is lower in developed nations at 2.2 per 100,000 compared with 4.3 per 100,000 in the developing nations [4].

In the European Union, about 34,000 new cases of cervical cancer related to HPV are diagnosed every year. The highest prevalence of cervical cancer associated with HPV infection are observed in Latvia (25.0/100,000 women), Bosnia and Herzegovina (23.9/100,000), and Estonia (22.5/100,000). On the other hand, the lowest prevalence is found in Malta (3.5/100,000), Switzerland (3.8/100,000), and Finland (4.7/100,000). In Poland, the prevalence is 9.4/100,000 women [9].

Currently, three vaccines are used in the world: 1. Cervarix (GlaxoSmithKline), a bivalent (2-V) vaccine targeting HPV16 and HPV18, the two most carcinogenic types; 2. Gardasil (Merck Inc., Meguro City, Tokyo), a quadrivalent (4-V) vaccine targeting HPV16/18 and the low risk types, HPV6 and HPV11, that cause genital warts; and 3. Gardasil 9 (Merck Inc.), a nonavalent (9-valent, 9-V) vaccine targeting HPV6/11/16/18 and the next five most carcinogenic types (HPV31/33/45/52/58). 

Most recommendations, including from the WHO, recommend routine HPV vaccination in girls aged 9–14 years before becoming sexually active. The secondary target group is girls over the age of 15 years and young women. HPV vaccination of males is currently not recommended as a priority [10,11]. However, the CDC recommends HPV vaccination at the age of 11 or 12 years (but it can start from the age of 9 years) and for men up to the age of 26 years, if not vaccinated already [12].

HPV vaccination was first introduced in 2007 in Australia and, since then, many countries have joined the vaccination program. In 2020, HPV vaccination has been introduced in 107 (55%) of the 194 WHO Member States. In the Americas and Europe, 85% and 77% of the countries have already introduced HPV vaccination. A lower number of countries with a HPV vaccination programme was observed in Oceania (56%), Asia (40%), and Africa (31%) [13].

The implementation of an HPV vaccine programme has created many controversies. The two most frequently cited sources of negative knowledge about HPV vaccination are the Canadian article “Guinea pigs” and the Danish paradocumentary “De Vaccinerede Piger” (The Vaccinated Girls—Sick and Abandoned). The article describes the vaccination programme as “the biggest Canadian science experiment in decades”. The paradocumentary tells the story of some girls who allegedly had a reaction to a HPV vaccination and suffered from POTS—postural orthostatic tachycardia syndrome. The paradocumentary presents the HPV vaccine in a very negative light, although no studies confirmed the described symptoms.

The vaccination coverage level depends on at what age the first dose of vaccination is being administered (higher coverage level in younger groups). However, in general, a high level of vaccination coverage was observed in Australia (78.6%) and in the United Kingdom (81%), in contrast with countries with low vaccination coverage level such as Georgia (36.2%), Lithuania (29%) [13], and Poland (7.5–10%) [14].

Until now, Poland was one of the EU countries where vaccination against HPV was recommended but not government-funded [14]. There are no restrictions to access of any type of HPV vaccine in Poland, but they are expensive. Difficulties limiting or even preventing the implementation of HPV vaccination in Poland include:

1. Lack of knowledge about HPV infections and vaccines.
2. Motivational obstacles for vaccination, including:
  a. lack of recommendations from the National Health Fund (NHF) and doctors,
  b. “bad attitude” towards vaccination in anti-vaccine environments,
  c. lack of support and conversations with parents about sexuality.
3. Logistical barriers, including:
  a. availability of vaccination,
  b. the price of the vaccine,
  c. the need to repeat vaccination (compliance).
4. Myths about the vaccine—mistaken beliefs, including:
  a. sexual promiscuity,
  b. negative information about the vaccine in the media (ineffective, not very well studied, dangerous).

Of these many barriers, the lack of support and conversations with parents about sexuality deserves special attention. This issue is critical because prophylactic vaccination of girls against HPV should be carried out in the period preceding sexual initiation. Therefore, the decision to vaccinate girls is the responsibility of parents who, by giving their consent, give them the chance to avoid a disease that is dangerous to their health and even their life when they become adults. 

The attitudes of parents and adolescents, using many research models [15,16,17], have already been the subject of many studies globally, including those conducted in Europe, and a review in this field was undertaken by Lopez et al. [18]. However, the situation in Poland was not considered in that review, so it is well-justified to present the results of studies that aim to assess: 1. The state of knowledge of parents about HPV infections and HPV vaccines; and 2. Parents’ attitude to vaccination, including HPV vaccines.

Cervical cancer remains high in incidence and mortality rankings in Poland. Despite this, HPV vaccines are not covered by the government. Many local governments in Poland organize free HPV vaccination programs, but HPV immunization remains low.

This study, the first of its kind to be undertaken in Poland, undertook an analysis to determine the barriers to vaccination acceptance. 

This study aimed to assess parents’ knowledge about HPV infections, assess parents’ knowledge about HPV vaccines, and assess the impact of parents’ knowledge about HPV infection and HPV vaccines on their attitude to primary prevention, i.e., vaccinations.

## 2. Materials and Methods

### 2.1. Design of the Study

An observational cross-sectional descriptive study was undertaken.

All subjects gave their informed consent for inclusion before they participated in the study. The study was conducted in accordance with the Declaration of Helsinki, and the protocol was approved by the Ethics Committee of the Medical University of Warsaw (AKBE/123/16).

### 2.2. Data Collection

The survey was conducted in 2018 in Warsaw, Poland, and included parents of the children admitted to the Department of Pediatric Dermatology at a multidisciplinary regional hospital.

Data was collected using questionnaires presented on electronic devices (such as tablets, laptops, mobile phones) during the admission of the patients to the Department of Pediatric Dermatology. A researcher who monitored the study and helped with technical problems was present to ensure that only one parent was involved in the study.

Four parents refused to fill in the questionnaire, answering “no time” as the reason. The study was completed when questionnaires from a minimum of 200 parents were obtained. None of the children of the surveyed parents were diagnosed with HPV-related lesions as a reason for admission to the department.

### 2.3. Sample Size

A goal of the study was to include as many participants as possible. At the initial stage, no formal calculation of sample size was carried out. The final number of 288 parents ensured precision (measured at half the length of the 95% confidence interval) of 4 percentage points for assessing a trait whose true prevalence was 50% (for which 50% is needed in the most significant sample to reach a particular precision value).

### 2.4. Statistical Analysis

The analysis was conducted mainly with the use of descriptive statistics. The results are presented in the form of frequency tables and cross tables. Statistical inference was performed using the chi-squared test or, in the case of low frequencies of the analyzed features, Fisher’s exact test.

Fisher’s exact test is used in the case of samples that are too small—when the observed values calculated with the chi-squared test are below 5. The calculations were performed using the statistics program R 3.5.1. All tests were performed at a significance level *p* = 0.05.

The parents’ responses regarding their knowledge about HPV were presented depending on their age, sex, their place of residence, and education. The parents’ knowledge about vaccination was also analyzed. The parents’ responses to the questions regarding their knowledge about vaccinations were classified as correct or incorrect. Total test result and results regarding the parents’ knowledge about HPV and HPV vaccine was expressed as a percentage of available points.

### 2.5. Questionnaire

The questionnaire survey (Appendix A) for parents consisted of 34 questions and was designed by the authors. It was preceded by preliminary information, which consisted of explaining the purpose of the study, details on how to contact the author, and information about the voluntary and anonymous nature of the survey.

The survey consisted of both single-choice and multiple-choice questions. The age question was an open-ended question. The rest of the questions were closed questions. Seven questions related to their knowledge about the HPV virus, and seven questions related to their knowledge about the HPV vaccine. The rest of the questions were about the parents’ attitudes to vaccination and demographic data. One hundred and eighty of the surveyed parents declared that they had heard about the HPV virus and filled in the entire questionnaire. The rest of the respondents answered “no” or “I do not know” and were asked to provide their age, gender, place of residence, and education.

## 3. Results 

### 3.1. Group Characteristics 

The study included the parents of the children admitted to the Department of Pediatric Dermatology in Warsaw, Poland. Two hundred and eighty-eight parents participated in the study. Most of the respondents were women (78.8%). Parents between the ages of 30 and 40 years accounted for 61% (n = 155) of the respondents, parents between the ages of 20 and 30 years accounted for 24% (n = 61), and parents between the ages of 40 and 70 years accounted for 15% (n = 38).

Furthermore, 38 parents (13.2%) lived in the countryside, 26 (9%) lived in a city with up to 20,000 inhabitants, 33 (11.5%) lived in a city with 20,000–100,000 inhabitants, 36 (12.5%) lived in a city with 100,000–500,000 inhabitants, and 155 (53.8%) lived in a city with more than 500,000 inhabitants.

Most of the respondents had higher education (71.2%, n = 205). Sixty-eight people had secondary education (23.6%), 13 people had basic vocational education (4.5%), and 2 people had primary education (0.7%). A summary of these data is presented in Table 1.

Among those surveyed, 180 declared that they had heard of the HPV virus and filled in the entire questionnaire. The characteristics differentiating these two groups are presented in Table 2. Most people declaring knowledge about HPV were women. The numbers of men who declared that they had heard about the virus (n = 33) and had not heard about the virus (n = 28) were similar.

### 3.2. Test Results

Table 3 provides a summary of the total test scores and the number and percentage of parents with a particular test score. The first division presents all the scores together with the frequency and percentage of parents who achieved them. The highest score was 86.5% of correct answers, and the lowest score was 21.6% of correct answers.

From an analysis of the second part of the table, 2.8% of the parents achieved a score of up to 30% of correct answers, 47.8% scored in the range of 31–60 (n = 86), and 49.4% scored in the range of 61–100 (n = 89). Therefore, as many as 50.6% of parents would not pass the test if the pass mark for this test was a score above 61% of correct answers.

Table 4 provides summary statements for the percentage of the parental test for HPV vaccination knowledge. The first division presents the test scores together with the frequency and percentage of parents who achieved them. The highest score was 92.3% of correct answers, and the lowest score was 23.1% of correct answers.

From an analysis of the second part of the table, 2.2% of parents achieved a score of up to 30% of correct answers, 32.8% scored in the range of 31–60, and 65.0% scored in the range 61–100. Therefore, 35% of parents would not pass the test if the pass mark for this test was a score above 61% of correct answers.

Table 5 contains the summary result of the parents’ test of knowledge about HPV infections. The first division presents the test scores together with the frequency and percentage of parents who achieved them. The highest test score was 84% of correct answers, and the lowest score was 16% of correct answers.

From an analysis of the second division, 6.7% of parents achieved a score of up to 30% of correct answers, 56.7% scored in the range of 31–60 (n = 102), and 36.7% scored in the range 61–100 (n = 66). Therefore, 63.4% of parents would not pass the test if the pass mark for this test was a score above 61% of correct answers.

### 3.3. Parents’ Knowledge—Correct Answers

Table 6 contains a summary list of answers to questions concerning parents’ knowledge about the HPV virus and HPV vaccinations. In this comparison, only 39.4% correctly indicated the association of HPV with cancers of the genitourinary organs, and 42.8% correctly indicated an association of HPV with papillary lesions of the genital organs. In addition, only 8.9% of parents indicated the answer “children” as a group exposed to HPV infection.

#### 3.3.1. Knowledge Regarding HPV

There were no significant statistical differences between the groups identified by the place of residence and the number of children. Considering the remaining characteristics (gender, age, education), parents with a higher education and women had more correct answers, which was statistically significant. Parents with a higher education had an overall higher test score and statistically more correct answers to questions about vaccine funding and vaccine availability in Poland and identified girls over the age of nine years as the target group for the vaccine. The number of correct answers decreased with the age of the parents.

#### 3.3.2. Knowledge Regarding Vaccine

Women scored a statistically significant higher percentage of correct answers regarding the availability of the vaccine in Poland and a statistically significant higher percentage of correct answers regarding vaccine funding and identified young girls before sexual initiation as the target group for the vaccine.

There were more statistically significant differences for incorrect vaccine responses between the education groups. People with a higher education had overall higher test scores and statistically answered more questions correctly about preventing or reducing the possibility of an HPV infection and about identifying the routes of transmission of the virus (through sexual intercourse, during natural childbirth). Women correctly identified most of the cervical cancer risk factors. The number of correct answers decreased with the age of the parents.

#### 3.3.3. Attitude towards Vaccination

The only factors differentiating the attitude towards vaccinations were knowledge and education. Parents with a test score in the third quartile indicated that the high effectiveness of the vaccine might influence their decision to vaccinate their children with the recommended vaccine. The remaining characteristics of the parents (sex, age, place of residence) did not significantly affect the attitude to vaccination. It was interesting that 55% of the parents would have vaccinated their children if the vaccination was covered by the government.

## 4. Discussion

This research is the first in Poland to assess the knowledge and attitudes of parents towards HPV vaccination. HPV vaccination may prevent diseases related to papillomaviruses, including cervical cancer. Research in this field has been conducted worldwide for many years [19,20,21,22,23,24,25,26,27,28,29,30,31,32,33], and an interesting analysis was conducted by Lopez et al. [18].

It should be emphasized, however, that the attitude of parents may be influenced by many factors, and one of the key influencing factors in Poland is the lack of recommendations and financing by the National Health Fund. 

According to a study by Gerend et al., people recommended by the NHF to be vaccinated against HPV were forty times more likely to get vaccinated [34]. This is also confirmed by other studies [35,36,37,38].

The lack of recommendation and funding may have a negative impact on parents’ attitudes, especially when the price of the vaccine is high. In these studies, over one-third of the respondents indicated the high price of the HPV vaccine as a possible reason for refusing to vaccinate their children.

However, it should be emphasized that in countries with recommendations and a reimbursement scheme, the problem of HPV vaccination is much more complex. As many as 28% of parents refuse to vaccinate in the USA, and 8% delay vaccination. The motivation of both groups for this type of behavior differs significantly. Parents who refused vaccinations gave reasons such as fear of promiscuity, lower vaccine efficacy expected, and higher expected side effects. Parents who delay vaccination do not rule out later vaccination after learning more about the vaccine [39]. In the study by Brabin et al., friends and school nurses (35% each) and teachers (20%) also influenced the views of girls about the HPV virus and HPV vaccination [40].

The state of parental knowledge has a crucial influence on the immunization of children. The study by Fishman et al. proved that mothers with more knowledge about HPV were not willing to vaccinate themselves or their daughters [41]. The present study found that the only factors that affect attitudes to vaccination are knowledge and education. The remaining characteristics of parents do not significantly affect the attitude to vaccination.

Lack of support from parents due to their fear of encouraging girls to engage in risky sexual behavior is also emphasized in the literature, reducing the motivation to vaccinate. Factors increasing parental acceptance of vaccination include HIV testing in the past, having an older daughter, having had more sexual partners, and having a family member with cancer [42]. The most common concerns indicated by parents in the available studies are related to vaccine safety, post-vaccination sexual promiscuity, moral problems related to sexuality, denial that the daughter is at risk, conservative and religious views, lack of knowledge, and unknown side effects [43].

An interesting aspect is a conversation with their children about HPV vaccinations as an introduction to sexuality. In the study by Marlow et al., mothers of the girls stated the age of their daughters at which they would like to start discussions with them about vaccinations, sex, cervical cancer, sexually transmitted diseases, HPV, and HPV vaccination (Table 7). The study shows that the age of the girls at which mothers would like to talk to them about HPV vaccines is statistically higher than the age of the girls at which mothers talk to them about general immunization, sex, and even higher for general discussions about HPV vaccines, the HPV virus, and sexually transmitted diseases [44].

The price of the vaccine is often discussed in the aspect of barriers that reduce vaccination. In the analysis, the low price of the vaccine was not a determinant for vaccinating children (0 answers). Most parents decided to vaccinate their child after a doctor recommended the vaccine or because the vaccine was very effective. However, in the case of HPV vaccination, more than one-third of the respondents indicated the high price of the HPV vaccine as a possible reason for not taking up vaccination (over two-thirds indicated possible side effects).

In the literature, concerns about the cost of the vaccine include costs to be borne by the parents and by healthcare professionals, and costs of the vaccine to be borne by the healthcare insurer [45]. Therefore, to increase vaccination availability in developing countries, vaccine companies have negotiated to lower the price of a single dose to 4.50–4.60 USD [46,47]. For comparison, in Poland, the price of a single dose of Cervarix and Silgard varies around 440 zloty for a single dose (around 110 USD). The price of the vaccine also affects the choice of the vaccine. Often, patients opt for a bivalent vaccine due to the lower cost.

This research is the first attempt in Poland to identify the most important barriers to the effective implementation of an HPV vaccination program, and thus to the prevention of diseases connected with this virus. The presented analysis may be of assistance in the implementation of HPV vaccination programs in Poland.

## 5. Conclusions

Parents’ knowledge of the HPV virus is insufficient and depends on sex, age, and education and is independent of place of residence and the number of children. The parents’ knowledge about the HPV vaccine is low and independent of the place of residence, the number of children, and depends on age, sex, and education. Further studies need to be carried out to provide information regarding pro-vaccine motivation tools.

## Figures and Tables

**Table 1:** Characteristic of the group.

Characteristic	Group Size	Options	N (%)
**Sex**	288	Women	227 (78.8%)
		Men	61 (21.2%)
**Age**	254	(20–30)	61 (24%)
		(31–40)	155 (61%)
		(41–70)	38 (15%)
**Education**	288	primary	2 (0.7%)
		vocational	13 (4.5%)
		secondary	68 (23.6%)
		higher	205 (71.2%)
**Residence**	288	countryside	38 (13.2%)
		city up to 20,000 inhabitants	26 (9.0%)
		city from 20,000 to 100,000 inhabitants	33 (11.5%)
		city from 100,000 to 500,000 inhabitants	36 (12.5%)
		city > 500,000 inhabitants	155 (53.8%)

**Table 2:** Characteristics of the group of parents divided into two groups, declaring knowledge about HPV and HPV vaccination and declaring the lack of knowledge.

			No Knowledge	Declaring Knowledge	*p*
**Age**	**<30**	**N**	23	38	0.073
		**%**	37.7	62.3	
	**(30–35)**	**N**	31	79	
		**%**	28.2	71.8	
	**(36–40)**	**N**	19	26	
		**%**	42.2	57.8	
	**(41–65)**	**N**	19	19	
		**%**	50	50	
**Sex**	**Women**	**N**	70	157	<0.001
		**%**	30.8	69.2	
	**Men**	**N**	33	28	
		**%**	54.1	45.9	
**Education**	**Rest**	**N**	54	29	<0.001
		**%**	65.1	34.9	
	**Higher**	**N**	49	156	
		**%**	23.9	76.1	
**Place of residence**	**countryside**	**N**	29	9	<0.001
		**%**	76.3	23.7	
	**city up to 20,000 inhabitants**	**N**	11	15	
		**%**	42.3	57.7	
	**city from 20,000 to** **100,000 inhabitants**	**N**	18	15	
		**%**	54.5	45.5	
	**city from 100,000** **to** **500,000 inhabitants**	**N**	11	25	
		**%**	30.6	69.4	
	**city > 500,000 inhabitants**	**N**	34	121	
		**%**	21.9	78.1	

**Table 3:** Summary test results.

Total Test Result (% of Correct Answers)	N	%
21.6	4	2.2%
29.7	1	0.6%
32.4	4	2.2%
35.1	5	2.8%
37.8	6	3.3%
40.5	6	3.3%
43.2	1	0.6%
45.9	10	5.6%
48.6	7	3.9%
51.4	14	7.8%
54.1	14	7.8%
56.8	5	2.8%
59.5	14	7.8%
62.2	13	7.2%
64.9	13	7.2%
67.6	13	7.2%
70.3	17	9.4%
73.0	10	5.6%
75.7	10	5.6%
78.4	5	2.8%
81.1	3	1.7%
83.8	3	1.7%
86.5	2	1.1%
**(% points)**		
(0–30)	5	2.8%
(31–60)	86	47.8%
(61–100)	89	49.4%

Total test result = overall knowledge points scored in test by parents. N = number of parents who achieved this test score; % = percentage of parents who achieved this test score.

**Table 4:** Summary test results—knowledge about HPV vaccine.

Knowledge about HPV Vaccine (% of Correct Answers)	N	%
23.1	4	2.2%
30.8	6	3.3%
38.5	8	4.4%
46.2	21	11.7%
53.8	24	13.3%
61.5	36	20.0%
69.2	37	20.6%
76.9	24	13.3%
84.6	13	7.2%
92.3	7	3.9%
**(% points)**		
(0–30)	4	2.2%
(31–60)	59	32.8%
(61–100)	117	65.0%

Total test result = overall knowledge points scored in test by parents; N = number of parents who achieved this test score; % = percentage of parents who achieved this test score.

**Table 5:** Summary test results—knowledge about HPV.

Knowledge about HPV (% of Correct Answers)	N	%
16.0	2	1.1%
20.0	4	2.2%
24.0	2	1.1%
28.0	4	2.2%
32.0	10	5.6%
36.0	1	0.6%
40.0	15	8.3%
44.0	20	11.1%
48.0	11	6.1%
52.0	7	3.9%
56.0	16	8.9%
60.0	22	12.2%
64.0	15	8.3%
68.0	21	11.7%
72.0	16	8.9%
76.0	8	4.4%
80.0	4	2.2%
84.0	2	1.1%
**(% points)**		
(0–30)	12	6.7%
(31–60)	102	56.7%
(61–100)	66	36.7%

Total test result = overall knowledge points scored in test by parents; N = number of parents who achieved this test score; % = percentage of parents who achieved this test score.

**Table 6:** Knowledge about the HPV virus and the HPV vaccine—correct answers to component questions.

Question	N	n (%)
**How can you get infected with HPV**		
By kissing	180	162 (90%)
By touch	180	24 (13.3%)
Sexual intercourse	180	138 (6.7%)
During natural childbirth	180	43 (23.9%)
By contact of infected blood with the blood of an uninfected person, e.g., using the same needle	180	142 (78.9%)
**Who is at risk of HPV infection?**		
Only women	180	146 (81.1%)
Only men	180	177 (98.3%)
Children	180	16 (8.9%)
Only homosexuals	180	180 (100%)
Both women and men, regardless of sexual orientation	180	142 (78.9%)
**HPV infection predisposes to:**		
Cancer of the genitourinary organs	180	71 (39.4%)
Cervical cancer	180	134 (74.4%)
Papillary lesions of the genital area	180	77 (42.8%)
**Does HPV infection always lead to the manifestation of the disease?**	180	134 (74.4%)
**Do you know what the purpose of the Pap smear test is?**	180	174 (96.7%)
**What factors increase the risk of developing cervical cancer?**		
Smoking cigarettes	180	54 (30%)
A family history of cervical cancer	180	120 (66.7%)
HPV infection	180	149 (82.8%)
A large number of sexual partners	180	107 (59.4%)
Lack of physical activity	180	173 (96.1%)
**How can HPV infection be prevented, or the risk of HPV infection be reduced?**		
By vaccination before sexual initiation	180	140 (77.8%)
By using condoms	180	114 (63.3%)
By limiting the number of sexual partners and by avoiding risky sexual behavior	180	127 (70.6%)
It is not possible to prevent HPV infection	180	178 (98.9%)
**Mean percentage result (S.D.)**	180	65.1 +/− 16.2
**Is there a vaccine against HPV?**	180	164 (91.1%)
**Is the HPV vaccine available in Poland?**	180	155 (86.1%)
**For which sex are HPV vaccines registered in Poland?**	180	124 (68.9%)
**The target groups for the vaccine are:**		
Girls around 12 years old	180	91 (50.6%)
Boys around 12 years old	180	165 (91.7%)
Young women before sexual initiation	180	98 (54.4%)
Young boys before sexual initiation	180	165 (91.7%)
Young women not infected with HPV	180	59 (32.8%)
Young men not infected with HPV	180	167 (92.8%)
The scientifically proven AEFI (Adverse events following immunization)	180	67 (37.2%)
**Does HPV vaccination give 100 percent protection?**	180	122 (67.8%)
**Is the cost of the vaccine in Poland covered by the government?**	180	81 (45%)
**Mean percentage result (S.D.)**	180	62.3 +/− 15.6

N = number of parents who answered the question.

**Table 7:** Average age of the discussion between parents and children on HPV-related topics (76).

Subject	Mean	S.D.	Statistical Difference between a Conversation about HPV Immunization and Other Topics of Conversation
Reason for vaccination	9.58	1.72	*t* = −24.46, *p* < 0.0001
Sex	10.61	1.73	*T* = −8.07, *p* < 0.0001
Cervical cancer	11.04	1.69	n.s. (not significant)
HPV vaccine	11.08	1.61	
HPV	11.18	1.60	*T* = 4.00, *p* < 0.0001
STDs	11.38	1.57	*T* = 7.27, *p* < 0.0001

## Data Availability

Not applicable.

## Appendix A

### Questionnaire

**1. Have you ever heard of the human papillomavirus (HPV) before?**

*Select one answer*

- Yes
- No
- I don’t remember

**2. How did you learn about HPV?**

*Select one answer*

- From a social campaign
- From a doctor
- From a leaflet in a medical facility
- From the press or TV
- From friends
- From the Internet

**3. How can you get infected with HPV?**

*You can tick several options*

- By kissing
- By touch
- Through sexual intercourse
- During natural childbirth
- By contact of infected blood with the blood of an uninfected person, e.g., using the same needle
- I do not know

**4. Who is at risk of HPV infection?**

*You can tick several options*

- Only women
- Only men
- Children
- Only homosexuals
- Both women and men, regardless of sexual orientation
- I do not know

**5. HPV infection predisposes to:**

*You can tick several options*

- Cancer of the genitourinary organs (vagina, penis, anus, vulva)
- Cervical cancer
- Head and neck cancer
- Papillary lesions of the genital area
- Respiratory papillomatosis
- I do not know

**6. Does HPV infection always lead to the manifestation of the disease?**

*Select one answer*

- Yes
- No
- I do not know

**7. Do you know what the purpose of the Pap smear test is?**

*Select one answer*

- Yes
- No
- I do not know

**8. What factors increase the risk of developing cervical cancer?**

- You can tick several options
- Smoking cigarettes
- A family history of cervical cancer
- HPV infection
- A large number of sexual partners
- Lack of physical activity
- I do not know

**9. How can HPV infection be prevented, or the risk of HPV infection be reduced?**

*You can tick several options*

- By vaccination before sexual initiation
- By using condoms
- By limiting the number of sexual partners and by avoiding risky sexual behavior
- It is not possible to prevent HPV infection
- I do not know

**10. Is there a vaccine against HPV?**

*Select one answer*

- Yes
- No
- I do not know

**11. Is the HPV vaccine available in Poland?**

*Select one answer*

- Yes
- No
- I do not know

**12. For which sex are HPV vaccines registered in Poland?**

*Select one answer*

- For women
- For men
- For both women and men
- I do not know

**13. The target groups for the vaccine in Poland are:**

*You can tick several options*

- Young women/girls around 12 years old
- Young men/boys around 12 years old
- Young women before sexual initiation
- Young men before sexual initiation
- Young women not infected with HPV
- Young men not infected with HPV
- All women, regardless of age
- All men, regardless of age
- I do not know

**14. The scientifically proven AEFI of HPV vaccination include:**

*Select one answer*

- Pain at the site of vaccination and fainting after vaccination
- Autism, ADHD and other central nervous system disorders caused by thiomersal (ethyl mercury compound used as a preservative in the vaccine)
- An anaphylactic reaction in children allergic to proteins connected with the cultivation of vaccine viruses in chicken embryos
- All of the above
- None of the above

**15. Does HPV vaccination give 100 percent protection against cervical cancer?**

*Select one answer*

- Yes
- No
- I don’t know

**16. Is the cost of the vaccine in Poland covered by the government?**

*Select one answer*

- Yes, 100%
- Yes, 50%
- Yes, but I do not know how much is covered by the government
- No
- I do not know

**17. What do you think about childhood vaccinations:**

*Select only one answer*

- I believe they are very much needed
- I believe they are unnecessary
- I consider them dangerous to health

**18. Do you have a child/children?**

*Select only one answer*

- Yes
- No

**19. Please enter the age of the child/children:**

*If you have more than one child, please state the age of all your children*

- Child 1:
- Child 2:
- Child 3:
- Child 4:
- Child 5:

**20. Please select the gender of your child (ren)**

*Please select only one answer per line*

- Male Female
- Child 1:
- Child 2:
- Child 3:
- Child 4:
- Child 5:

**21. Have you vaccinated your child (ren) with obligatory vaccinations?**

*Select only one answer*

- Yes, I have vaccinated them with all obligatory vaccinations
- Yes, but only with selected vaccinations
- No, neither
- I do not remember

**22. Why did you not vaccinate your/their child/children?**

*Select only one answer*

- Because vaccinations are dangerous to your health
- I did not know about such an obligation
- I forgot and the doctor didn't remind me
- Difficult access to medical services

**23. Did you vaccinate your child (s) with the vaccines recommended but optional (additionally payable)?**

*Select only one answer*

- Yes
- No
- I do not remember

**24. What influenced your decision to vaccinate your child/you with the vaccine recommended but optional?**

*Select multiple answers*

- Opinion about the vaccine on the Internet
- Vaccine safety
- Low price of the vaccine
- The vaccine is highly effective
- Positive doctor's statement about the vaccine
- Opinion of friends about the vaccine

**25. Please select the opinion with which you agree.**

*Select multiple answers*

- Vaccinating my child against HPV may contribute to unsafe sexual behavior
- Having my child vaccinated against HPV may contribute to prior sexual initiation
- It would be good to talk to your child about the HPV vaccine as an introduction to a conversation about human sexuality
- I do not agree with any of the above opinions

**26. Have you vaccinated your children against HPV?**

*Select only one answer*

- Yes
- No

**27. What influenced your decision to vaccinate your child/you with the vaccine against HPV?**

*Select multiple answers*

- Opinion about the vaccine on the Internet
- Vaccine safety
- Low price of the vaccine
- The vaccine is highly effective
- Positive doctor's statement about the vaccine
- Opinion of friends about the vaccine

**28. Are you willing to vaccinate your children against HPV? Select only one answer**

- Yes
- No
- I do not know yet

**29. What factors may influence your decision not to vaccinate yours? Children against HPV?**

*Select multiple answers*

- The high price of the vaccine
- Side effects of the vaccine
- Fear that the vaccine may cause children to engage in risky behavior sexual
- Reluctance to make children aware of human sexuality
- I believe this vaccine is unnecessary
- I believe this vaccine is ineffective
- I believe this vaccine is dangerous to health

**30. Would you vaccinate your child against HPV if vaccination was covered by the government?**

*Select only one answer*

- Yes
- No
- I do not know

**31. Please enter your gender:**

*Select only one answer*

- Woman
- Man

**32. Please enter your age:**

**33. Please provide your education:**

*Select only one answer*

- Primary
- Vocational
- Secondary
- Higher

**34. Size of the place of residence**

*Select only one answer*

- village
- city up to 20,000 inhabitants
- city from 20,000 up to 100,000 inhabitants
- city from 100,000 up to 500,000 inhabitants
- city above 500,000 inhabitants

**35. How old are you?**

**36. Please select your gender:**

- Female
- Male
